# Improving the quality of steamed bread with whole soybean pulp: Effects of ultrasonic treatment on protein structure and reduction of beany flavor

**DOI:** 10.1016/j.ultsonch.2024.107156

**Published:** 2024-11-29

**Authors:** Feng Han, Jialin Song, Mingming Qi, Yueming Li, Mei Xu, Xin Zhang, Chuangshuo Yan, Shanfeng Chen, Hongjun Li

**Affiliations:** School of Agricultural Engineering and Food Science, Shandong University of Technology, Zibo 255049, Shandong, China

**Keywords:** High-intensity ultrasound, Whole soybean pulp, Steamed bread, Quality, Protein structure, Beany flavor

## Abstract

Incorporation of whole soybean pulp (WSP) into wheat flour has been shown to improve the nutritional profile of steamed bread. However, this substitution often disrupts the protein network and introduces an undesirable beany flavor, compromising the overall quality of the steamed bread. This research explored the impacts of varying ultrasonic power levels on the quality of steamed bread containing WSP (WSPSB), with the goal of improving both the protein network structure and the flavor profile. The findings indicated that at an ultrasonic power of 300 W, WSPSB had an 18.10 % decrease in hardness and a 14.93 % increase in specific volume compared to the 0 W. Results from CLSM, SDS-PAGE, fluorescence intensity, surface hydrophobicity, and FTIR spectroscopy revealed that ultrasonic treatment modified the secondary protein structure by increasing the proportion of β-sheets and random coils. These changes facilitated better integration of soybean protein and gluten, thereby strengthening the steamed bread’s protein network. Furthermore, analyses of volatile flavor components, molecular docking, and correlation studies indicated that alterations in the protein structure mitigated the binding of beany flavor components to proteins, leading to significant reductions in their presence—specifically, a 7.12 % decrease in 1-Octen-3-ol and an 8.47 % decrease in Furan, 2-pentyl-. Overall, ultrasound treatment effectively refined the protein network and mitigated the beany flavor in steamed bread, thereby improving its quality.

## Graphical abstract

**Figure:**
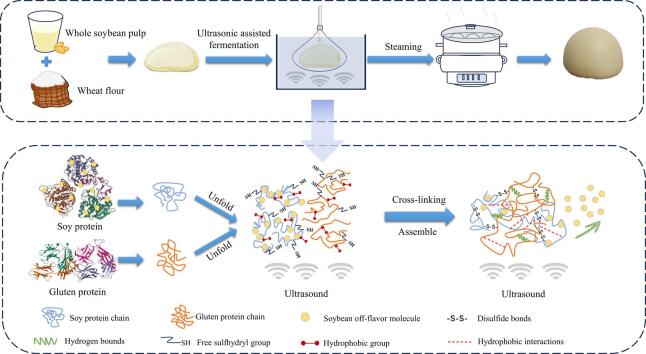

## 1 Introduction

The primary components of Chinese steamed bread (CSB) include refined wheat flour, yeast, and water. The refinement process significantly reduces the nutritional content of wheat flour, stripping away dietary fiber, protein, vitamins, polyphenols, and minerals [1], [2]. Soybeans, a staple crop in China, offer a nutrient-rich alternative for the food industry, containing essential amino acids like lysine, dietary fiber, phenolic acids, and isoflavones [3], [4], [5]. Substituting partial of wheat flour with soybeans could address the deficiency of lysine in wheat flour and enhance steamed bread’s nutritional profile. Typically, soy product preparation, including soy flour and soy protein, involves complex processing steps such as dehulling, separation, and drying [3]. However, using a hot water milling process for soaked soybeans to create whole soybean pulp (WSP) can streamline these steps, reduce energy use, and lessen environmental impact, while ensuring complete utilization of the soybeans.

While partially replacing wheat flour with soybean can improve the nutritional value of steamed bread, excessive soy inclusion can disrupt the gluten network formation, negatively affecting the steamed bread’s quality [6]. Furthermore, the beany flavor associated with soybeans significantly limits consumer acceptance. This flavor primarily arises from the oxidative degradation of lipids during processing, with beany flavor compounds tending to bind to proteins via non-covalent interactions [7], [8]. Consequently, there is a critical need to explore techniques that both improve the protein network and reduce the beany flavor in steamed bread incorporating whole soybean pulp (WSPSB).

High-intensity ultrasound, a novel physical technology, is widely used in food processing owing to its safe, non-toxic, and eco-friendly properties [9]. It operates primarily through three mechanisms: cavitation, mechanical, and thermal effects [10]. Several researches have studied the impact of ultrasonic treatment on dough’s gluten network structure and beany flavor content. For example, studies by Cao et al. and Zhang et al. demonstrated that ultrasound could enhance the protein network in dough, thereby improving the quality of food products [11], [12]. Similarly, Song et al. and Kong et al. found that ultrasound disrupted interactions among protein molecules, altered protein conformation, and affected protein aggregation, significantly influencing how beany flavor compounds bind to proteins [13], [14]. Despite these insights, there is limited study specifically focused on the effect of ultrasound on the protein network formation and beany flavor in whole soybean pulp steamed bread (WSPSB).

This experiment assessed the effect of varying ultrasonic powers on the quality, protein network, and beany flavor of whole soybean pulp steamed bread (WSPSB). The assessments involved texture analysis, confocal laser scanning microscopy (CLSM), SDS-PAGE, Fourier-transform infrared spectroscopy (FTIR), free amino acid content, and volatile flavor compound profiling. Additionally, molecular docking and correlation analysis were used to investigate the relationship between alterations in protein structure and beany flavor in WSPSB. These findings offer a theoretical framework for understanding how ultrasonic treatment influences the protein network structure and beany flavor of WSPSB.

## 2 Materials and methods

### 2.1 Materials

Commercial wheat flour, containing 10.96 % moisture, 10.68 % protein, and 74.15 % starch, were procured from Yunhai Flour factory, Zibo, China. Yeast (Angle Yeast Co., Ltd., China), and soybean (Jiaodong Soybean No.1) were procured from a local farmer’s market in Zibi, China. Only round, complete, and bright yellow soybeans were selected. After that, the soybeans were rinsed and left to soak in water at room temperature (RT, 24 ± 1 °C) for 12 h. To make WSP, the soaked soybeans were ground in the XZ-3 high-speed blender (Xinze Electrical Appliances Co, Ltd, China) for 3 min at 33,000 r/min with boiled water (the dry soybeans to water ratio was 1:3, w/v).

### 2.2 Preparation of steamed bread

The WSP was incorporated into wheat flour at a 40 % substitution rate (composite flour basis). The steamed bread were prepared using the procedure in accordance with the earlier study [12]. Initially, wheat flour, WSP, and 1 % active dry yeast (based on the weight of wheat flour and WSP) were pre-mixed. Water was added according to farinographic absorption rates, which were 57.5 mL for the control steamed bread (CSB) and 15 mL for the 40 % WSP flour mixture per 100 g of flour. The mixture was hand-kneaded for 3 min. The control dough underwent an initial fermentation at 32 °C and 80 % relative humidity for 60 min in a BRF-18C fermentation chamber (Guangzhou Zhanzhuo Commercial Equipment Manufacturing Co., Ltd., Guangzhou, China). Initial fermentation phase of the 40 % WSP dough: the dough with 40 % WSP was put in a plastic bag with a pipette to ensure gas exchange, and then sonicated for 40 min at 32 °C using different ultrasonic powers in an ultrasonic cleaner (0 W, 200 W, 300 W, 400 W, and 500 W). Following the initial fermentation, the dough was pressed 10 times on the sheeter, and then kneaded into a round shape. Second fermentation phase of the dough: the kneaded dough was further fermented at 32 °C and 80 % relative humidity for 15 min. Finally, the fermented dough was steamed for 20 min, waited for 3 min in the steamer, and then cooled at room temperature (RT, 24 ± 1 °C) for 50 min. 0 W, 200 W, 300 W, 400 W, and 500 W were used to indicate the WSPSB samples under different ultrasonic intensity, respectively.

### 2.3 Steamed bread qualities

#### 2.3.1 Specific volume

Millet displacement method was applied to determine the specific volume of the steamed bread [15].

#### 2.3.2 Texture profile analysis (TPA)

The Texture Analyzer (TA-XT Plus, Stable Micro-systems, UK), equipped with a P/36R probe and a 5 kg loading cell, was applied to evaluate the steamed bread. The device was set with pre-test, test, and post-test speeds of 1.0, 1.0, and 2.0 mm/s, respectively. All measurements were performed in triplicate.

#### 2.3.3 Color parameters

The color of the steamed bread crust and crumb was analyzed via a colorimeter (CM-3600A, Konica Minolta, Osaka, Japan). The color parameters recorded were redness/greenness (a*), yellowness/blueness (b*), and lightness (L*). Each measurement was performed quadruplicate. The total color difference (ΔE) indicated the color change of the steamed bread at different ultrasonic power levels compared to the control steamed bread (CSB), and was calculated as the formula below:

(1) $$\Delta E=\sqrt{{{(a}^{*}-a_{0}^{*})}^{2}+{{(b}^{*}-b_{0}^{*})}^{2}+{{(L}^{*}-L_{0}^{*})}^{2}}$$

where a *, b * and L * are the color parameters of the steamed bread with WSP added; a_0_*, b_0_* and L_0_* are the color parameters of the CSB.

#### 2.3.4 Steamed bread structure evaluation

The structure of the samples was evaluated following Atudorei’s method with slight modifications [16]. The microstructure of steamed bread crumb was observed using A Leica TL3000 Ergo stereomicroscope (Leica, Shanghai, China) at a magnification of 47 × and a resolution of 4000 × 3000 pixels.

### 2.4 Confocal laser scanning microscopy (CLSM)

Steamed bread were cut into slices (40 μm thick) and then stained with Rhodamine B (0.5 %, w/v) and Fluorescein 5-isothiocyanate (FITC, 0.3 %, w/v) to mark proteins (red) and starch (green) in the samples. Proteins and starch were stained for 10 min and 20 min, respectively. The stained samples were then observed under the laser scanning confocal microscopy (LSM 900, Carl Zeiss AG, Germany) with an Olympus inverted microscope (20 × ). Proteins and starch were visualized at excitation wavelengths of 568 nm and 488 nm.

### 2.5 Free SH and disulfide bond (S-S) content

The free SH and S-S contents in freeze-dried steamed bread samples were measured using Ellman's reagent DTNB [17].

### 2.6 Protein particle size distribution

Protein solutions were prepared based on Han’s method [18]. The steamed bread samples with 1 g of protein were dispersed in acetic acid solution (50 mL, 0.5 mol/mL) after magnetic stirring for 2 h, and then centrifuged at 10,000 r/min for 20 min. The proteins’ particle size distribution in the supernatant was measured by a laser particle size analyzer (Mastersizer 2000, Malvern Co. Ltd., UK).

### 2.7 Sodium dodecyl sulphate–polyacrylamide gel electrophoresis (SDS-PAGE)

SDS-PAGE analysis was performed in accordance with Xu’s method [19]. Steamed bread powder (19 mg) was dispersed in buffer solution (1 mL), thoroughly mixed, incubated in a water bath at 30 °C for 3 h, centrifuged at 10,000 r/min for 15 min, and the supernatant was boiled for 5 min. For non-reducing electrophoresis, the buffer contained 10 % (v/v) glycerol, 2 % (v/v) SDS, 0.063 mol/L Tris-HCl, and 0.01 % (v/v) bromophenol blue. For reducing conditions, 10 % (v/v) β-mercaptoethanol was added to the buffer. A 20 μL sample was loaded onto the stacking gel (4 %, w/w), followed by protein separation using the separating gel (10 %, w/w).

### 2.8 Intrinsic fluorescence spectroscopy

The fluorescence spectra of samples was analyzed on the basis of Han’s method [14]. Steamed bread powder (15 mg) was dispersed in 15 mL of phosphate buffer (pH 7), magnetically stirred for 4 h, and centrifuged at 4500 r/min for 20 min. The supernatant was measured with a spectrofluorometer (RF-6000, Shimadzu, Japan).

### 2.9 Surface hydrophobicity (H_0_)

The H_0_ of the steamed bread was measured based on Song’s method [13]. Steamed bread samples (300 mg) were dispersed in phosphate buffer (pH 7) and centrifuged for 20 min at 4500 r/min after shaking in a water bath for 1 h at 30 °C. H_0_ was analyzed using 1-anilino-8-naphthalenesulfonate (ANS) as a fluorescent probe.

### 2.10 Fourier transform infrared (FTIR) spectroscopy

Steamed bread powder was mixed with potassium bromide (KBr) and compressed into pellets. The secondary structure of the steamed bread was measured via FTIR spectroscopy. The samples were scanned over the wavenumber range of 400–4000 cm^−1^ with a resolution of 4 cm^−1^ and an average of 32 scans.

### 2.11 Free amino acids (FAAs) content

1 g of steamed bread powder was diluted with HCl (25 mL, 0.01 M) and extracted by ultrasonication for 1 h at 40 °C, followed by centrifugation at 4500 r/min for 20 min. The supernatant was combined with an equal volume of 10 % 5-Sulfosalicylic acid dihydrate and left to stand at 4 °C for 40 min before being centrifuged at 12,000 r/min for 30 min. The free amino acids in the final supernatant were analyzed using a High-Speed amino acid analyzer (LA8080, Hitachi, Ltd., Japan)

### 2.12 Volatile flavor compounds

The volatile organic compound profiles of steamed bread were examined using an Agilent 8890 GC system equipped with 5977B MS detector (Agilent Technologies Co., Ltd., Santa Clara, USA). Steamed bread samples (1.5 g) were placed in headspace bottles (20 mL). It was equilibrated in a water bath at 60 °C for 15 min and then extracted using SPME fiber at 60 °C for 40 min. After desorption at the GC inlet for 5 min, the assay was carried out at 250 °C.

### 2.13 Molecular docking

The interaction between soybean proteins and flavor molecules was investigated using a molecular docking method. The PDB ID of soy protein 11S was 1FXZ, and the PDB ID of soy protein 7S was 3AUP. The PubChem database provided the crystal structures of Furan, 2-Pentyl (CID: 19602), and 1-Octen-3-ol (CID: 18827). The software AutoDock Vina 1.1.2 was used to carry out the molecular docking.

### 2.14 Statistical analysis

Each analysis was performed in triplicate (n = 3). All data were presented as mean ± standard deviation. The means were assessed by analysis of variance (ANOVA) and compared using Duncan's test (p < 0.05). R 4.4.0 software was chosen to draw heat maps and to make Pearson's correlation analysis.

## 3 Results and discussion

### 3.1 Steamed bread qualities

#### 3.1.1 Specific volume and TPA

The specific volume and soft texture of steamed bread are critical factors influencing consumer preference. Table 1 demonstrates the impacts of varying ultrasound powers on the specific volume and texture of WSPSB. The steamed bread’s specific volume decreased from 2.7 mL/g to 2.01 mL/g with the substitution of WSP, indicating that WSP diluted the gluten content in the flour and hindered the formation of gluten network [20]. However, after ultrasound-assisted fermentation, the steamed bread’s specific volume increased markedly (p < 0.05). Notably, at 300 W ultrasound power, the specific volume increased by 14.93 % in comparison with the 0 W, aligning with the findings of Nouska [21]. This result revealed that ultrasonic treatment enhanced the protein network’s structural stability, thereby increasing the specific volume of the steamed bread.

**Table 1:** Specific volume and texture of steamed bread under different ultrasonic power.

Sample	Specific volume (mL/g)	Texture				
		Hardness (g)	Springiness (mm)	Cohesiveness	Chewiness (g.mm)	Resilience
CSB	2.69 ± 0.01^a^	1316.19 ± 18.15^e^	0.94 ± 0.01^a^	0.85 ± 0.01^a^	1092 ± 32.01^c^	0.49 ± 0.01^ab^
0 W	2.01 ± 0.03^e^	4677.00 ± 83.62^a^	0.91 ± 0.03^ab^	0.81 ± 0.01^a^	3457.44 ± 76.77^a^	0.48 ± 0.01^b^
200 W	2.22 ± 0.01^bc^	4205.73 ± 128.11^c^	0.92 ± 0.1^ab^	0.87 ± 0.03^a^	3365.53 ± 13.04^a^	0.52 ± 0.02^ab^
300 W	2.31 ± 0.02^b^	3830.44 ± 38.78^d^	0.92 ± 0.01^ab^	0.87 ± 0.05^a^	3047.51 ± 174.35^b^	0.53 ± 0.03^a^
400 W	2.19 ± 0.03^c^	4095.93 ± 105.35^c^	0.92 ± 0.02^ab^	0.84 ± 0.02^a^	3172.60 ± 180.88^ab^	0.52 ± 0.00^ab^
500 W	2.12 ± 0.04^d^	4390.07 ± 31.01^b^	0.89 ± 0.01^b^	0.85 ± 0.02^a^	3322.84 ± 140.17^ab^	0.50 ± 0.01^ab^

Values within the same column followed by different letters were significantly different (p < 0.05).

The effect of varying ultrasound power levels on the steamed bread’s physical characteristics was evaluated by TPA. Compared with CSB, the 0 W sample showed significantly higher hardness and chewiness, along with significantly lower springiness, cohesiveness, and resilience (p < 0.05), which was ascribed to the disruption of the steamed bread protein network structure by WSP. However, at 300 W ultrasound power, the steamed bread’s hardness and chewiness decreased from 4677.00 g and 3457.44 g.mm to 3830.44 g and 3047.51 g.mm, respectively, while the resilience increased from 0.48 to 0.53 compared to the 0 W, which indicated that ultrasound could improve the WSPSB’s quality. Therefore, ultrasound-assisted fermentation is effective in fostering the formation of a stable protein network and improving the overall quality of steamed bread.

#### 3.1.2 Color

The effect of varying ultrasonic power levels on the color of steamed bread is shown in Table 2. The substitution of WSP to wheat flour resulted in steamed bread that was darker, redder, and yellower. The pigments in soybeans were the primary contributors to the color of WSPSB [16]. Furthermore, Protonotariou et al. discovered that an optimal protein network structure in steamed bread was associated with increased brightness and decreased red and yellow color values [22]. In the 0 W sample, the addition of WSP disrupted the gluten network structure of WSPSB compared to CSB, leading to higher a* and b* values and lower L* values. After ultrasonic treatment, the a*, b*, and ΔE values markedly decreased, while the L* values of the steamed bread markedly increased (p < 0.05). This improvement in color could be ascribed to ultrasound promoting protein aggregation, thereby enhancing the visual quality of the steamed bread.

**Table 2:** Crust color and crumb color of steamed bread under different ultrasonic power.

Sample	Crumb				Crust			
	L*	a*	b*	ΔE	L*	a*	b*	ΔE
CSB	83.93 ± 0.58^a^	−0.44 ± 0.03^d^	15.01 ± 0.24^c^	−	84.86 ± 0.37^a^	−0.42 ± 0.01^d^	17.11 ± 0.23^c^	−
0 W	76.89 ± 0.60^d^	0.86 ± 0.04^a^	21.99 ± 0.59^a^	10.00 ± 0.74^a^	80.74 ± 0.20^c^	0.77 ± 0.08^b^	25.51 ± 0.37^a^	9.43 ± 0.42^a^
200 W	80.48 ± 0.26^bc^	0.56 ± 0.00^b^	20.83 ± 0.38^b^	6.85 ± 0.19^bc^	81.15 ± 0.08^c^	0.63 ± 0.05^c^	24.59 ± 0.07^b^	8.42 ± 0.08^b^
300 W	81.04 ± 0.52^b^	0.34 ± 0.02^c^	20.35 ± 0.10^b^	6.13 ± 0.28^c^	81.83 ± 0.17^b^	0.60 ± 0.10^c^	24.49 ± 0.57^b^	8.05 ± 0.48^b^
400 W	79.88 ± 0.36^c^	0.34 ± 0.06^c^	20.84 ± 0.60^b^	7.15 ± 0.60^bc^	81.17 ± 0.04^c^	0.61 ± 0.00^c^	24.75 ± 0.13^b^	8.55 ± 0.12^b^
500 W	79.64 ± 0.25^c^	0.49 ± 0.07^b^	20.91 ± 0.45^b^	7.36 ± 0.47^b^	81.06 ± 0.09^c^	1.05 ± 0.02^a^	26.07 ± 0.19^a^	9.84 ± 0.14^a^

Values within the same column followed by different letters were significantly different (p < 0.05).

#### 3.1.3 Steamed bread structure evaluation

**Fig. 1:**
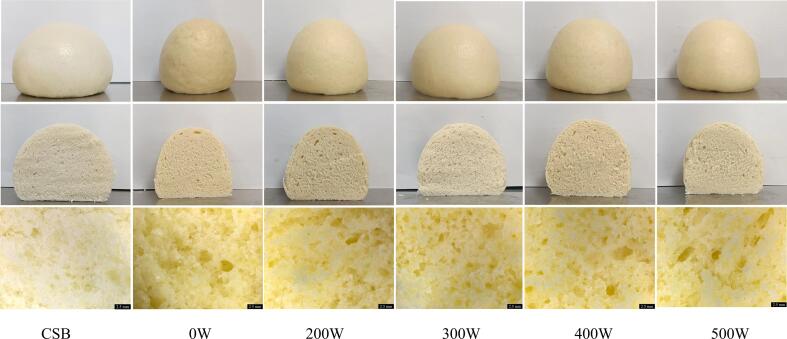
Appearance and crumb microstructure of steamed bread under different ultrasonic power.

The impacts of varying ultrasonic power levels on the appearance and internal microstructure of steamed bread are shown in Fig. 1. The 0 W sample had a rough surface, larger average pore sizes, and lower pore density with the addition of WSP. These findings revealed that the addition of WSP had a negative impact on the protein network structure and reduced the air-holding capacity of the steamed bread. In contrast, the ultrasonically treated steamed bread exhibited a smooth and intact surface, with smaller and more uniform crumb pores compared to the 0 W. This suggested that ultrasonic treatment strengthened the stability of the protein network structure, leading to improve air-holding capacity in the steamed bread [23].

### 3.2 CLSM analysis

**Fig. 2:**
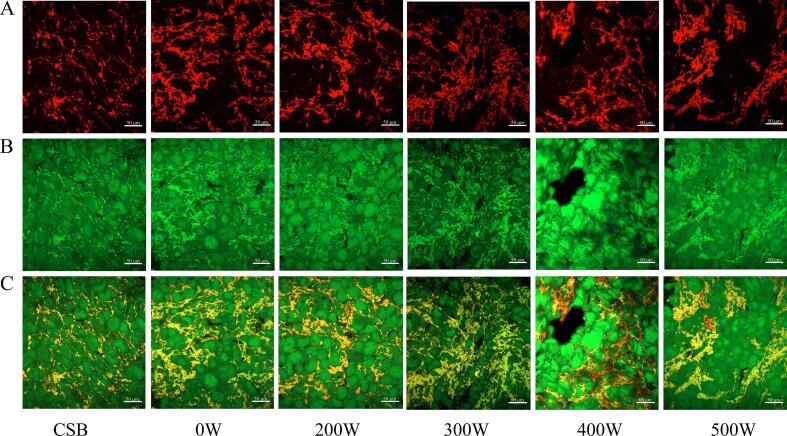
Microstructure and distribution of steamed bread protein and starch under different ultrasound intensities (C). The protein (A) and starch granules (B) were stained red and green, respectively. (For interpretation of the references to color in this figure legend, the reader is referred to the web version of this article.)

CLSM was implemented to analyze the microstructure changes in steamed bread under different ultrasound power levels. As shown in Fig. 2, the protein network was stained red by Rhodamine B, and the starch was stained green by FITC. The CSB in Fig. 2 presented a homogenous and continuous protein network structure with starch granules evenly embedded in the protein network structure. In contrast, the 0 W sample displayed larger gaps, and a coarser, discontinuous protein network structure, demonstrating that the addition of WSP diluted the gluten content, and hindered the formation of an effective protein network. Notably, after ultrasound-assisted fermentation, the WSPSB’s protein network structure appeared more homogeneous and continuous in comparison with the 0 W sample, indicating that ultrasonic treatment facilitated protein aggregation and improved the protein network structure. As ultrasound power increased from 0 W to 300 W, the protein network structure of steamed bread progressively improved, with the most significant enhancement observed at 300 W. However, excessive ultrasonic power (> 300 W) led to disrupted protein aggregation, resulting in a weaker and discontinuous protein network. In summary, the ultrasound’s cavitation effect altered the conformation of soy and gluten proteins, promoting their aggregation and enhancing the formation of the protein network in WSPSB [24], [25].

### 3.3 Protein particle size distribution analysis

**Fig. 3:**
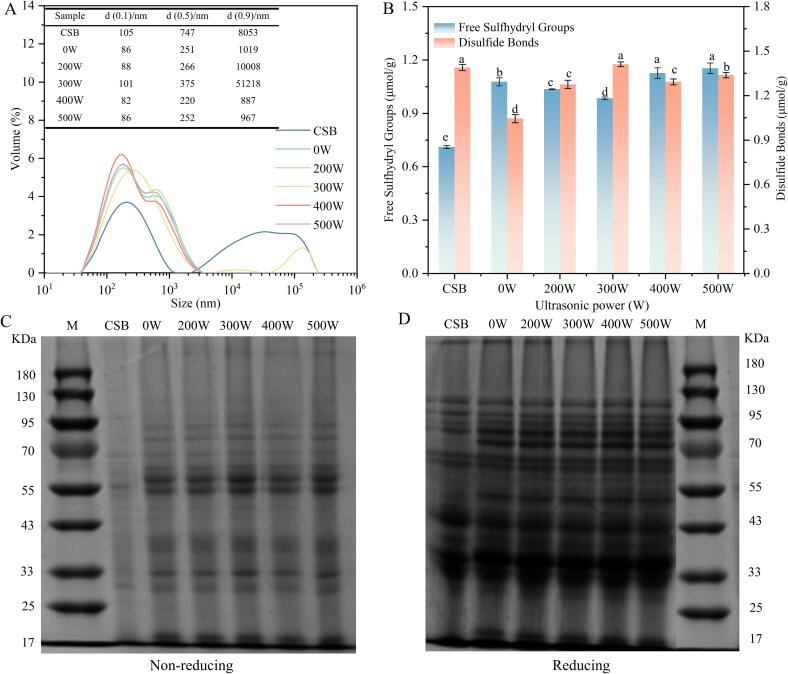
Effect of different ultrasonic power levels on the microstructure of steamed bread. (A) Protein particle size distribution; (B) Free sulfhydryl and disulfide bond content; (C) Electropherograms under reducing conditions (SDS-PAGE); (D) Electropherograms under non-reducing conditions (SDS-PAGE). Lane M represents the molecular marker. Different letters (a-d) indicate significant differences between values (P < 0.05).

The impact of varying ultrasonic power levels on protein aggregation in steamed bread was further analyzed by measuring the protein particle size. Fig. 3A shows the impact of varying ultrasound powers on the protein particle size distribution in steamed bread. Compared to CSB, the 0 W sample exhibited a protein particle size distribution ranging from 10 nm to 3500 nm, with no significant peak beyond 3500 nm, indicating that the addition of WSP disrupted gluten protein aggregation and weakened the protein network structure in WSPSB. After ultrasound-assisted fermentation, the particle size distribution of WSPSB shifted significantly to the right, and a small peak emerged near 1 × 10^5^ nm when the ultrasound power was set at 300 W. Additionally, the median protein particle diameter (d(0.5)) in the ultrasonicated samples followed a trend of initially increasing and then decreasing, with the 300 W sample reaching a median diameter of 375 nm. These findings suggest that appropriate ultrasonic treatment (300 W) induced protein unfolding, promoting protein aggregation, and finally increasing the protein particle size in WSPSB [26].

### 3.4 Free SH and S-S analysis

Free SH groups and S-S play an essential role in stabilizing protein structure [27]. The interactions between proteins under different ultrasonic powers were investigated by measuring the content of free SH and S-S in samples. As shown in Fig. 3A, the 0 W sample had significantly lower S-S content (p < 0.05) and significantly higher free SH content (p < 0.05) compared to CSB. This was likely due to the addition of WSP, which diluted the gluten proteins and disrupted the disulfide bonds in the wheat gluten system, leading to increased exposure of SH groups [25]. Ultrasonic treatment altered the contents of free SH and S-S in the WSPSB. At 300 W ultrasound power, the free SH content significantly decreased while the S-S content notably increased in comparison with the 0 W sample (p < 0.05). This suggested that ultrasonic treatment promoted cross-linking of cysteine SH groups, enhanced protein interactions, and facilitated the formation of a more ordered protein network [24]. In addition, ultrasound-induced generation of –OH radicals from water molecules can oxidize SH groups to form S-S bonds, further stabilizing the protein network [11]. Therefore, optimal ultrasonication at 300 W facilitated the conversion of SH groups to S-S bonds, enhancing the stability of the protein network in steamed bread.

### 3.5 SDS-PAGE analysis

Non-reducing and reducing SDS-PAGE analyses were conducted to evaluate the impact of ultrasonic power on the interaction between gluten and soy proteins. Fig. 3C and D demonstrate the protein migration patterns under non-reducing and reducing conditions, respectively. In Fig. 3C, the 0 W sample revealed the aggregation of 11S and 7S soy proteins, resulting in the appearance of new bands around 90, 75, 48, and 33 k Da [28]. In addition, the intensity of the 0 W bands on non-reduced SDS-PAGE was significantly enhanced, and new electrophoretic bands appeared in the range of approximately 55 K Da bands compared to CSB. These changes suggested that soy proteins interacted with gluten proteins to form protein polymers [25]. Compared to the 0 W sample, ultrasound treatment intensified the electrophoretic bands in WSPSB, likely due to the cavitation and mechanical effects of ultrasound, which altered protein structures and promoted the aggregation of soy proteins with gluten proteins [29].

In the reduced SDS-PAGE, where disulfide bonds are disrupted by β-mercaptoethanol. Fig. 3D shows that the intensity of the electrophoretic bands in the 40 % WSP sample was enhanced compared to CSB. Gluten proteins are rich in tyrosine, which can be catalyzed by peroxidases to form tyrosyl cross-links, contributing to the structural stability of the protein network in addition to disulfide bonds [30], [31]. This indicated that soy proteins might cross-link with gluten proteins through both disulfide bonds and tyrosyl groups. Moreover, ultrasound-assisted fermentation further intensified the band intensity in WSPSB, likely due to ultrasound-induced alterations in gluten protein structure that exposed tyrosine residues and promoted cross-linking between soy and gluten proteins, contributing to a stable protein network [32]. Therefore, ultrasound effectively promoted the aggregation of soy and gluten proteins, enhancing the stability of the protein network structure in WSPSB.

### 3.6 Intrinsic fluorescence spectroscopy analysis

**Fig. 4:**
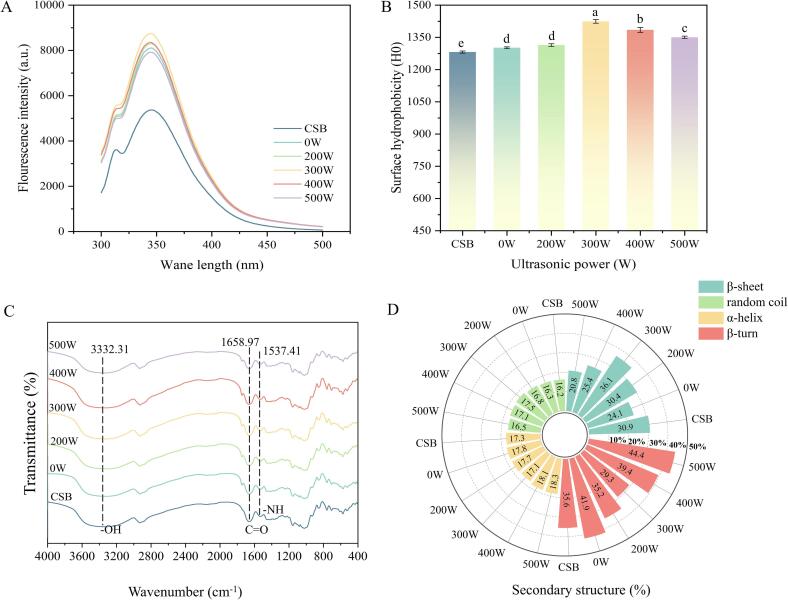
Fluorescence spectra (A), H_0_ (B), FTIR spectra (C) and secondary structure content (D) of steamed bread treated with different ultrasonic power levels. Different letters (a-d) indicate significant differences between values (P < 0.05).

Amino acid residues are stabilized through hydrophobic interactions, ionic interactions, and hydrogen bonding to form a stable three-dimensional protein conformation [33]. Changes in protein conformation during ultrasonic treatment can be evaluated by analyzing the fluorescence spectra of aromatic amino acid residues. The fluorescence spectroscopy results for ultrasound-assisted fermented steamed bread are shown in Fig. 4A. The 0 W sample exhibited significantly higher fluorescence intensity than CSB (p < 0.05), likely owing to the abundance of aromatic amino acids in soybeans. Furthermore, as the ultrasonic power increased, the fluorescence intensity of the steamed bread samples exhibited a tendency of increasing and then decreasing, reaching a peak at 300 W. Song et al. demonstrated that ultrasonic treatment could alter the spatial arrangement and interactions between amino acids [13]. This pattern suggested that the ultrasound’s cavitation effect promoted protein unfolding and exposure of chromophore groups, thereby influencing protein structure.

### 3.7 H_0_ analysis

H_0_ is crucial for maintaining protein conformational stability and function. The impact of varying ultrasonic power levels on the structure and function of steamed bread proteins was evaluated by determining the H_0_ of samples treated at varying ultrasound power levels (Fig. 4B). Ultrasound-assisted fermentation markedly increased the H_0_ of the steamed bread in comparison with the 0 W sample. This suggests that the ultrasound’s cavitation effect promoted the unfolding of protein molecules, exposing hydrophobic groups that were originally hidden within the protein structure. Nevertheless, excessive ultrasound power (> 300 W) disrupted the hydrophobic core, reducing the binding sites for ANS [13], [14]. In summary, applying ultrasonic treatment during dough fermentation induced the exposure of hidden hydrophobic groups and altered protein conformation.

### 3.8 FTIR analysis

As shown in Fig. 4C, FTIR spectroscopy was applied to reveal the effect of ultrasound on the protein secondary structure and chemical bonding in steamed bread. The peaks at 1658.97 cm^−1^ (Amide I), 1537.41 cm^−1^ (Amide II), and 3332.31 cm^−1^ correspond to C = O stretching, N-H bending, and O-H stretching vibrations, respectively [34], [35]. The 0 W sample exhibited lower absorption intensities in the Amide I, Amide II, and 3332.31 cm^−1^ bands compared to the CSB, which was likely owing to the disruption of the gluten network by WSP. After ultrasound-assisted fermentation, the absorption intensities of these bands increased, suggesting that ultrasound could modify the protein secondary structure, promote hydrogen bond formation, and enhance interactions between soy and gluten proteins. Nevertheless, excessive ultrasonic power (>300 W) disrupted these protein interactions, compromising the stability of the protein network structure.

The amide I region (1600–1700 cm^−1^) was investigated to study protein secondary structure, where 1600–1640 cm^−1^ is β-sheet, 1640–1650 cm^−1^ is random coil, 1650–1660 cm^−1^ is α-helix, and 1660–1700 cm^−1^ is β-turn [36]. Fig. 4D presents the calculated relative contents of the protein secondary structures in steamed bread. Compared to CSB, the 0 W sample showed a 22.01 % decrease in β-sheet content and a 17.70 % increase in β-turn content, likely due to the disruption of the gluten protein network by WSP, affecting the stability of protein conformation. After ultrasound-assisted fermentation, the relative contents of random coil and β-sheet initially increased and then decreased, while the opposite trend was found in β-turn and α-helix. The increase in β-sheet content supports protein aggregation and network formation, while the decrease in α-helix content is associated with disulfide bond formation and protein aggregation between soy and gluten [37]. At 300 W ultrasound power, the relative contents of random coil and β-sheet reached their maximum values, indicating that this power level most effectively promoted the formation and stabilization of the protein network in WSPSB.

### 3.9 FAAs analysis

**Fig. 5:**
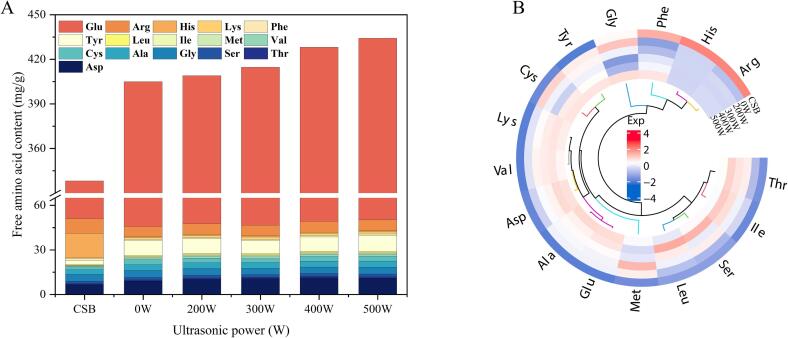
Free amino acid content (A) and heat map of free amino acid content (B) in steamed bread subjected to different ultrasonic power levels.

Fig. 5A and B illustrate the levels of 16 free amino acids in steamed bread, with the data normalized to generate a heat map. Due to the absence of gluten proteins in WSP and its disruption of the gluten network when added to wheat flour, the 0 W sample exhibited higher free amino acid content than CSB. Ultrasound-assisted fermentation influenced the levels of free amino acids in WSPSB. At 300 W ultrasound power, the levels of Ser, Gly, Cys, Met, Tyr, and Arg were the lowest. The trends observed in sulfur-containing amino acids (Cys and Met) and Tyr were consistent with the disulfide bond and SDS-PAGE results, indicating that ultrasound facilitated the aggregation of soy proteins with gluten proteins, thereby reducing the free amino acid content in WSPSB. Additionally, free amino acids contribute to the sensory quality of steamed bread [38]. The content of bitter amino acids (Met, Tyr, and Arg) in the samples (300 W) decreased after ultrasonic treatment compared with the 0 W sample. The conclusion revealed that ultrasound could improve the protein network and enhance the sensory quality of steamed bread.

### 3.10 Volatile flavor compounds analysis

**Fig. 6:**
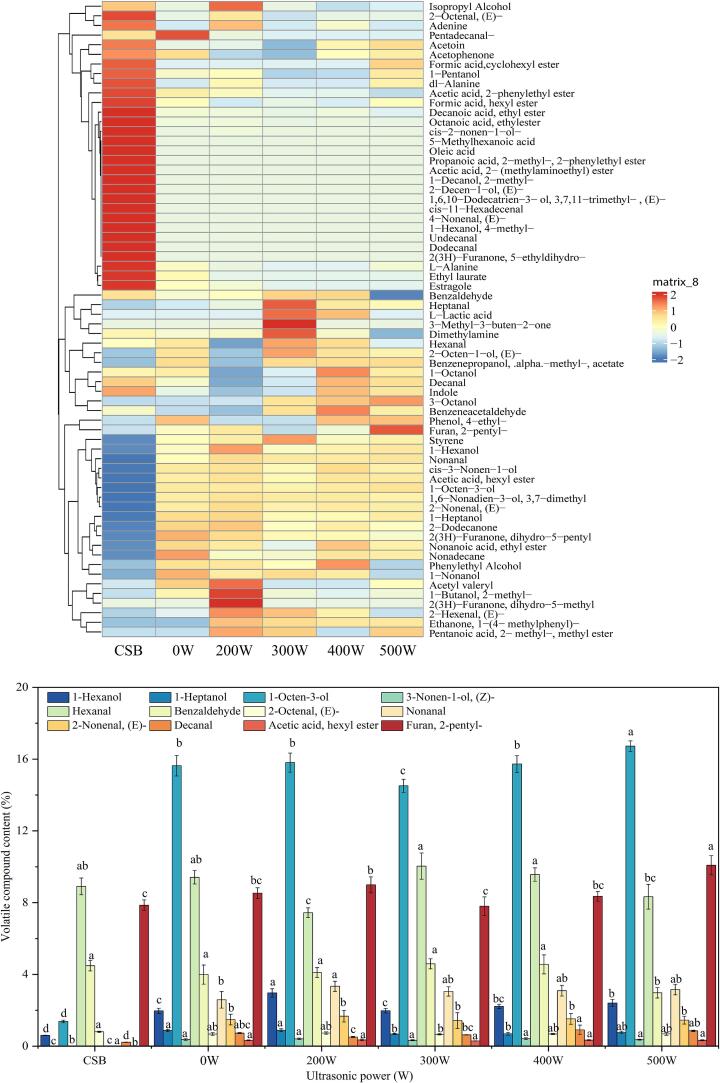
Heat map of volatile flavor compounds (A) and relative content of major volatile beany flavor compounds (B) in steamed bread treated with different ultrasonic power levels.

The beany flavor negatively impacts the sensory properties of WSPSB, significantly affecting consumer acceptance. As shown in Table S1, a total of 67 volatile flavor compounds were identified across all steamed bread samples, including 18 alcohols, 14 aldehydes, 6 ketones, 16 esters, 5 acids, and 8 other compounds, with alcohols, aldehydes, and esters constituting the largest proportions. The primary beany flavors include notes such as mushroom, grassy, green, earthy, fatty, and soapy [19]. As illustrated in Fig. 6B, 12 key volatile flavor components representing beany flavors were selected based on Table S1 and Fig. 6A to assess the impact of ultrasonic treatment on these off-flavors. Compared to the 0 W, the levels of 1-Hexanol, 1-Heptanol, 1-Octen-3-ol, 3-Nonen-1-ol (Z-), 2-Octenal (E-), Nonanal, 2-Nonenal (E-), Decanal, acetic acid hexyl ester, and Furan, 2-pentyl- were reduced when the ultrasonic power was set to 300 W. This result suggested that moderate ultrasonic power could effectively reduce the beany flavor in WSPSB. Ultrasonic treatment likely enhanced the binding between soy and gluten proteins, disrupting the interaction between soy protein and flavor compounds, thereby mitigating the beany flavor in the samples [39]. However, higher ultrasonic power (> 300 W) led to an increase in the content of these beany flavor compounds, likely because excessive ultrasound disrupted the aggregation of soy and gluten proteins, exposing internal hydrophobic regions, which in turn enhanced the binding affinity between flavor compounds and proteins [36]. Notably, the aldehyde content (e.g., Hexanal and Benzaldehyde) increased in the samples treated at 300 W compared to the 0 W sample, possibly due to ultrasound-induced partial lipid oxidation, which elevated aldehyde levels [39]. Overall, ultrasonic treatment proved effective in reducing the beany flavor of WSPSB.

### 3.11 Molecular docking and correlation analysis

**Fig. 7:**
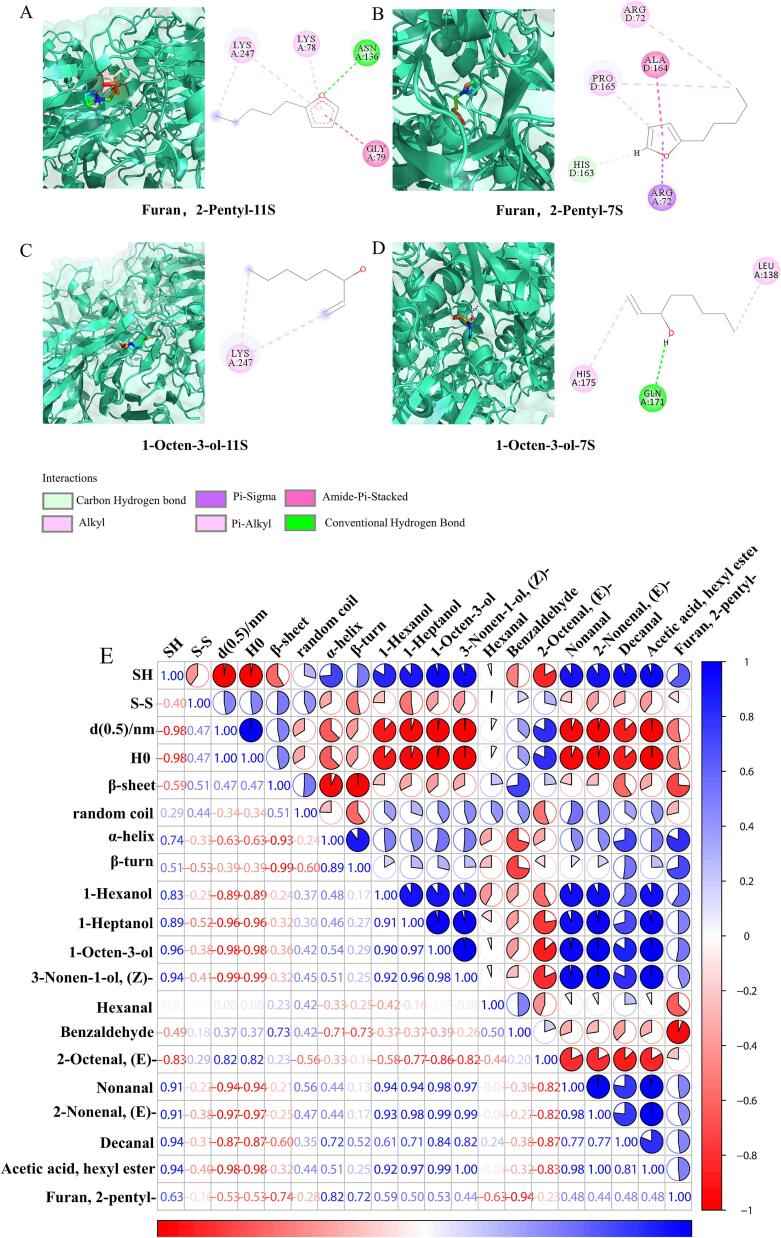
Optimal molecular docking models of Furan, 2-Pentyl- with 11S protein (A) and 7S protein (B), and 1-Octen-3-ol with 11S protein (C) and 7S protein (D). Correlation between protein structure and beany flavor in steamed bread treated with different ultrasonic power levels (E).

To gain insights into the interaction patterns between soy proteins and volatile flavor molecules, Furan, 2-pentyl- and 1-Octen-3-ol, which are closely associated with beany flavor, were selected for docking with the 11S and 7S soy proteins. The binding energies of Furan, 2-pentyl- and 1-Octen-3-ol to 11S and 7S proteins, as shown in Table S2, were −4.9, −5.2, −4.5, and −4.6 kcal/mol, respectively—values well below the threshold of −1.20 kcal/mol, indicating reliable molecular docking results. Fig. 7A and B display the optimal 3D and 2D docking conformations of Furan, 2-pentyl- with the 11S and 7S proteins. The docking analysis revealed that Furan, 2-pentyl- interacted with both 11S and 7S proteins through hydrophobic interactions and hydrogen bonding [40]. Similarly, Fig. 7C and D illustrate the 3D and 2D docking positions of 1-Octen-3-ol with the 11S and 7S proteins. 1-Octen-3-ol was found to bind to 11S primarily through hydrophobic interactions and to 7S through a combination of hydrophobic interactions and hydrogen bonding [41]. These findings reveal that flavor molecules interact with soy proteins mainly through hydrophobic interactions and hydrogen bonding. The cavitation and mechanical effects of ultrasound could induce changes in protein conformation. Moderate ultrasonication (300 W) enhanced the interaction between soy and gluten proteins, reduced binding sites between soy proteins and flavor compounds, and significantly decreased the beany flavor of WSPSB (p < 0.05), as evidenced by SDS-PAGE, fluorescence intensity, H_0_, and FTIR spectroscopy results.

Pearson correlation analysis was performed to further explore the connection between protein structure and flavor characteristics in steamed bread following ultrasound-assisted fermentation. As shown in Fig. 7E, there was a remarkable correlation between volatile flavor compounds and the protein structure of the steamed bread. After ultrasound-assisted fermentation, disulfide bonds, particle size, and H_0_ showed a positive correlation with β-sheet content and a negative correlation with α-helix content. This indicated that ultrasound-assisted fermentation enhanced protein structure ordering, promoted the aggregation of soy and gluten proteins, and strengthened the protein network in the steamed bread. Additionally, the levels of 1-Hexanol, 1-Heptanol, 1-Octen-3-ol, 3-Nonen-1-ol (Z-), 2-Octenal (E-), Nonanal, 2-Nonenal (E-), Decanal, acetic acid hexyl ester, and Furan, 2-pentyl- were negatively correlated with disulfide bonds, particle size, H_0_, and β-sheet content, while showing a positive correlation with SH groups, random coil, and α-helix content. These researches reveal that ultrasound-assisted fermentation modified the conformations of soy and gluten proteins, promoted protein aggregation, and disrupted protein interactions with beany flavor compounds, ultimately reducing the number of binding sites for these undesirable flavors. In summary, ultrasound-assisted fermentation is a viable method for improving the protein network structure and mitigating the beany flavor in steamed bread.

## 4 Conclusion

This study examined the impacts of varying ultrasonic power levels on the quality, structure, and flavor of WSPSB. Ultrasonic treatment enhanced the quality of WSPSB, resulting in a smooth surface and a crumb with small, uniform pores. The CLSM images, increased protein particle size, higher S-S content, and SDS-PAGE results demonstrated that 300 W ultrasonic treatment effectively promoted protein aggregation and improved the protein network structure in the steamed bread. Additionally, ultrasound-assisted fermentation induced structural and conformational changes in the proteins, leading to increased fluorescence intensity and H_0_, higher relative content of random coil and β-sheet structures, and decreased relative content of β-turn and α-helix structures. Analysis of volatile flavor compounds, along with molecular docking and correlation analysis, revealed that 300 W ultrasonic treatment decreased the relative content of beany flavor in WSPSB. This reduction was likely owing to changes in protein structure and the aggregation of soy and gluten proteins, which lowered the binding sites for beany flavor compounds. In conclusion, ultrasound is an effective approach for improving the quality of WSPSB while reducing their beany flavor, which is essential for expanding the application of WSP in the food industry.

## CRediT authorship contribution statement

**Feng Han:** Conceptualization, Methodology, Investigation, Writing – original draft, Writing – review & editing. **Jialin Song:** Investigation. **Mingming Qi:** Investigation. **Yueming Li:** Investigation. **Mei Xu:** Investigation. **Xin Zhang:** Investigation. **Chuangshuo Yan:** Investigation. **Shanfeng Chen:** Writing – review & editing. **Hongjun Li:** Conceptualization, Writing – review & editing, Project administration, Funding acquisition.

## Declaration of competing interest

The authors declare that they have no known competing financial interests or personal relationships that could have appeared to influence the work reported in this paper.
