# Supplementary material for: Improving the quality of steamed bread with whole soybean pulp: Effects of ultrasonic treatment on protein structure and reduction of beany flavor
Source: Ultrason Sonochem. 2024 Nov 29;112:107156. doi: 10.1016/j.ultsonch.2024.107156 (PMC11647651; doi:10.1016/j.ultsonch.2024.107156)
Supplement: Supplementary Data 1 [file mmc1.docx]

Table.S1. Concentrations of volatile flavor compounds in steamed bread after ultrasound-assisted fermentation. Flavor was obtained from Flavor Ingredient Library (<https://www.femaflavor.org/flavor>) and PubChem (<https://pubchem.ncbi.nlm.nih.gov/>).

| Compounds | Flavors | Relative amount (%) | | | | | |
| --- | --- | --- | --- | --- | --- | --- | --- |
|  |  | CSB | 0W | 200W | 300W | 400W | 500W |
| Alcolols |  |  |  |  |  |  |  |
| Isopropyl Alcohol | Burnt, Cocoa, Floral, Malt | 1.33 | 0.49 | 1.82 | 0.48 | 0.16 | 0.32 |
| 1-Pentanol | Green, wax | 6.37 | 4.77 | 4.91 | 3.94 | 4.03 | 5.24 |
| 1-Hexanol | Banana, Flower, Grass, Herb | 0.60 | 1.97 | 2.97 | 1.98 | 2.22 | 2.40 |
| 1-Heptanol | Musty, rancid | - | 0.87 | 0.89 | 0.67 | 0.69 | 0.77 |
| 1-Octen-3-ol | Cucumber, Earth, Fat, Floral, Mushroom | 1.38 | 15.63 | 15.81 | 14.51 | 15.73 | 16.72 |
| cis-2-nonen-1-ol- | Melon aroma | 4.40 | - | 0.14 | 0.04 | - | - |
| 1-Octanol | Bitter Almond, Burnt Matches, Fat, Floral | 0.41 | 0.42 | 0.29 | 0.35 | 0.49 | 0.42 |
| Phenylethyl Alcohol | Mild, warm,rose, honey-like odour | 4.49 | 6.78 | 6.63 | 6.04 | 7.61 | 4.78 |
| 1-Nonanol | Fat, Floral, Green, Oil | 0.06 | 0.21 | 0.17 | 0.19 | 0.17 | 0.09 |
| 1-Decanol, 2-methyl- | - | 0.28 | - | - | - | - | - |
| 1,6,10-Dodecatrien-3- ol, 3,7,11-trimethyl- , (E)- | Fir, Linoleum, Pine | 0.27 | - | - | - | - | - |
| 1-Butanol, 2-methyl- | cooked roasted aroma with fruity or alcoholic undertones | - | 0.14 | 0.36 | 0.08 | 0.04 | 0.08 |
| 3-Octanol | Citrus, Moss, Mushroom, Nut, Oil | - | 0.01 | 0.02 | 0.22 | 0.27 | 0.32 |
| cis-3-Nonen-1-ol | Floral, Green, Pungent | - | 0.38 | 0.41 | 0.33 | 0.42 | 0.36 |
| 2-Octen-1-ol, (E)- | Meaty, roasted aroma | - | 0.11 | - | 0.15 | 0.11 | 0.09 |
| 1,6-Nonadien-3-ol, 3,7-dimethyl | floral | - | 0.87 | 0.94 | 0.88 | 0.88 | 0.84 |
| 2-Decen-1-ol, (E)- |  | 0.65 | - | - | - | - | - |
| 1-Hexanol, 4-methyl- | Sweet | 1.11 | - | - | - | - | - |
| total | - | 21.35 | 32.65 | 35.36 | 29.86 | 32.82 | 32.43 |
| Aldehydes |  |  |  |  |  |  |  |
| Hexanal | Fresh, green, fatty, aldehydic, grass, leafy, fruity, sweaty | 8.91 | 9.41 | 7.44 | 10.04 | 9.57 | 8.33 |
| Heptanal | Dry fish | 0.03 | 0.26 | 0.53 | 2.07 | 1.15 | 0.88 |
| Benzaldehyde | Bitter Almond, Burnt Sugar, Cherry, Malt, Roasted Pepper | 4.48 | 3.99 | 4.10 | 4.59 | 4.56 | 2.97 |
| Benzeneacetaldehyde | Berry, Geranium, Honey, Nut, Pungent | 0.38 | 0.29 | 0.25 | 0.46 | 0.55 | 0.43 |
| 2-Octenal, (E)- | Fatty, green aroma | 0.80 | 0.68 | 0.73 | 0.66 | 0.68 | 0.68 |
| Nonanal | Fat, Floral, Green, Lemon | - | 2.58 | 3.34 | 3.05 | 3.09 | 3.16 |
| 2-Nonenal, (E)- | Powerful, penetrating fatty, violet aroma | - | 1.48 | 1.67 | 1.42 | 1.52 | 1.45 |
| Decanal | Floral, Fried, Orange Peel, Penetrating, Tallow | 0.88 | 0.73 | 0.51 | 0.63 | 0.91 | 0.85 |
| Pentadecanal- | - | 0.61 | 1.18 | 0.14 | 0.01 | 0.01 | 0.01 |
| Undecanal | Sweet, fatty, floral odour | 0.31 | - | - | - | - | - |
| Dodecanal | fatty odour | 0.17 | - | - | - | - | - |
| cis-11-Hexadecenal | - | 0.39 | - | - | - | - | - |
| 2-Hexenal, (E)- | Strong fruity, green, vegetable-like aroma | - | 0.10 | 0.51 | 0.42 | 0.29 | 0.04 |
| 4-Nonenal, (E)- | Fruity aroma | 1.75 | - | - | - | - | - |
| total |  | 18.71 | 20.70 | 19.22 | 23.35 | 22.33 | 18.80 |
| Ketones |  |  |  |  |  |  |  |
| Acetoin | Buttery odor； Bland, woody, yogurt odor | 13.70 | 11.09 | 11.03 | 9.86 | 11.95 | 12.55 |
| Acetophenone | Sweet, pungent and strong medicinal odor | 0.08 | 0.06 | 0.01 | - | 0.04 | 0.05 |
| 2-Dodecanone | - | - | 0.20 | 0.21 | 0.14 | 0.17 | 0.14 |
| Ethanone, 1-(4- methylphenyl)- | Strong fruity-floral, warm, sweet odour | - | - | 0.10 | 0.09 | 0.08 | 0.08 |
| Acetyl valeryl | Buttery odour | - | 0.02 | 0.03 | - | - | 0.01 |
| 3-Methyl-3-buten-2-one | Pleasant, pungent, sweet | - | - | - | 0.01 | - | - |
| total |  | 13.78 | 11.37 | 11.38 | 10.10 | 12.24 | 12.83 |
| Esters |  |  |  |  |  |  |  |
| Acetic acid, 2- (methylaminoethyl) ester |  | 0.45 | - | - | - | - | - |
| Formic acid, hexyl ester | Ethereal, fruity, leafy, green odour | 18.23 | 13.06 | 10.76 | 11.64 | 10.92 | 13.04 |
| Acetic acid, hexyl ester | Sweet-fruity, pearl-like odor | - | 0.32 | 0.35 | 0.29 | 0.34 | 0.33 |
| 2(3H)-Furanone, dihydro-5-methyl | Warm, sweet, herbaceous odour | - | - | 0.11 | - | - | - |
| Benzenepropanol, .alpha.-methyl-, acetate | Mild, green, fruity odour | - | 0.02 | - | 0.02 | 0.02 | 0.02 |
| Propanoic acid, 2-methyl-, 2-phenylethyl ester | Fruity-rosy odour | 0.44 | - | - | - | - | - |
| Octanoic acid, ethylester | Wine, brandy, fruity floral odour | 10.33 | 1.78 | 1.80 | 1.38 | 1.64 | 1.35 |
| 2(3H)-Furanone, 5-ethyldihydro- | Herbaceous, sweet odour | 0.17 | - | - | - | - | - |
| Formic acid,cyclohexyl ester | Pleasant cherry-like odour | 0.10 | - | - | - | - | 0.06 |
| Acetic acid, 2-phenylethyl ester | Fruity odor | 1.31 | 0.86 | 0.75 | 0.59 | 0.60 | 0.48 |
| Nonanoic acid, ethyl ester | Fatty, fruity brandy-like odour | - | 0.16 | 0.14 | 0.08 | 0.17 | 0.13 |
| 2(3H)-Furanone, dihydro-5-pentyl | Strong odor reminiscent of coconut | - | 0.35 | 0.31 | 0.24 | 0.24 | 0.20 |
| Decanoic acid, ethyl ester | Oily brandy-like odour | 3.54 | 1.02 | 0.95 | 0.79 | 0.94 | 0.63 |
| Ethyl laurate | Fruity, floral odour | 0.23 | 0.06 | - | - | - | - |
| Pentanoic acid, 2- methyl-, methyl ester | - | - | - | 0.06 | 0.05 | - | 0.05 |
| Formic acid, heptyl ester | - | - | - | - | - | - | - |
| total |  | 34.35 | 17.63 | 15.23 | 15.08 | 14.87 | 16.29 |
| Acids |  |  |  |  |  |  |  |
| L-Alanine | Odorless | 0.15 | 0.03 | 0.01 | - | - | 0.02 |
| dl-Alanine | Odorless | 0.82 | - | 0.29 | - | - | 0.34 |
| 5-Methylhexanoic acid | Cheese fermented odour | 0.52 | - | - | - | - | - |
| Oleic acid | Acid, Fruit, Pungent, Sour, Vinegar | 0.14 | - | - | - | - | - |
| L-Lactic acid |  | - | - | - | 0.76 | 0.54 | - |
| total |  | 1.63 | 0.03 | 0.30 | 0.76 | 0.54 | 0.36 |
| Other |  |  |  |  |  |  |  |
| Dimethylamine | Ammonia- or fish-like odor | 0.42 | 0.33 | 0.35 | 0.77 | 0.35 | 0.06 |
| Styrene | Aromatic odor | 0.08 | 6.36 | 7.43 | 10.52 | 6.13 | 6.93 |
| Phenol, 4-ethyl- | sweet odour | - | 0.04 | - | - | 0.04 | 0.04 |
| Furan, 2-pentyl- | Fruity aroma | 7.86 | 8.53 | 8.99 | 7.80 | 8.35 | 10.08 |
| Adenine |  | 0.07 | 0.03 | 0.06 | 0.03 | 0.04 | 0.03 |
| Estragole | odor of anise | 0.25 | 0.07 | 0.05 | 0.02 | 0.02 | 0.02 |
| Indole | Unpleasant odor; Pleasant odor in dilute solutions | 1.03 | 0.71 | 0.55 | 0.64 | 0.99 | 0.90 |
| Nonadecane | Fuel-like | 0.47 | 1.54 | 1.07 | 1.05 | 1.29 | 1.23 |
| total |  | 10.18 | 17.61 | 18.50 | 20.83 | 17.21 | 19.69 |

Table S2. ligand (Furan，2-Pentyl and1-Octen-3-ol) and soy protein (7S and 11S) docking findings.

|  | binding energy (kcal/mol) | hydrogen bond | Hydrophobic interaction |
| --- | --- | --- | --- |
| Furan，2-Pentyl-11S | -4.9 | Asn-A:136 | Lys-A:247, Lys-A:78, Gly-A:79 |
| Furan，2-Pentyl-7S | -5.2 | His-D:163, | Arg-D:72, Ala-D:164, Pro-D:165, Arg-A:72 |
| 1-Octen-3-ol-11S | -4.5 | - | Lys-A:247 |
| 1-Octen-3-ol-7S | -4.6 | Gln-A:171 | His-A:175, Leu-A:138 |
